# Rabbit Carcasses for Use in Feline Diets: Amino Acid Concentrations in Fresh and Frozen Carcasses With and Without Gastrointestinal Tracts

**DOI:** 10.3389/fvets.2020.592753

**Published:** 2021-01-21

**Authors:** Tammy J. Owens, Andrea J. Fascetti, C. Christopher Calvert, Jennifer A. Larsen

**Affiliations:** ^1^Department of Molecular Biosciences, School of Veterinary Medicine, University of California, Davis, Davis, CA, United States; ^2^Department of Animal Science, College of Agricultural and Environmental Sciences, University of California, Davis, Davis, CA, United States

**Keywords:** rabbit, amino acids, nutrition, feline, taurine, carcass, rabbit (lagomorph), prey

## Abstract

Whole-prey diets for exotic feline species are common, and this practice has also increased in popularity for domestic cats. However, prior analyses of prey indicate possible essential amino acid inadequacy, and dilated cardiomyopathy from taurine deficiency was reported in cats fed whole ground rabbit. Crude protein, body water, and amino acid concentrations were evaluated in fresh and frozen ground rabbits with (*n*=10) or without (*n* = 10) gastrointestinal tracts. Amino acids were greater in fresh samples without gastrointestinal tracts (*p* < 0.05) except taurine, glycine, and cysteine. When normalized for protein content, only glutamate, alanine, methionine, isoleucine, tyrosine, lysine, histidine, and arginine were greater in fresh rabbits without gastrointestinal tracts (g/16 g N basis; *p* < 0.05). Freezing at −18°C for 30 days had no effect on crude protein or body water content. After freezing, only methionine was lower and only proline was higher when gastrointestinal tracts were omitted (g/16 g N basis; *p* < 0.05). Regardless, all essential amino acids except taurine exceeded Association of American Feed Control Officials and National Research Council nutrient recommendations for all feline life stages. In contrast, there was minimal impact of treatment on taurine concentrations. However, although feline taurine requirements for prey and other raw or fresh food diets remain undefined, none of the rabbit samples met any recommendation for taurine concentrations for commercial canned or dry extruded diets, ranging from 20 to 90% of the minimum values. Taurine supplementation is recommended when feeding rabbit to cats. Determination of taurine requirements of cats fed whole-prey diets is warranted.

## Introduction

The domestic cat (*Felis silvestris catus*) has been a successful hunter throughout its evolution and typically consumes a wide range of small prey (1–4). Accordingly, compared to dogs, cats have increased protein and amino acid (AA) requirements, differences in carbohydrate metabolism, and certain nutrients with limited endogenous synthesis remain essential (5). In particular, taurine deficiency can lead to dilated cardiomyopathy (DCM), reproductive abnormalities, and central retinal degeneration in both domestic and exotic felids (5–7). Whole-prey diets are increasing in popularity for domestic cats (8, 9) and are common for captive exotic felids. However, taurine requirements, known to vary by diet type, remain undefined for whole-prey diets. Greater than 50% of required dietary taurine replaces taurine degraded by intestinal microbes (5); however, many dietary and processing factors influence the amount required (10–20). Rabbits are a popular whole-prey item; however, information on nutrient composition is limited or conflicting (8, 9, 21–24), and DCM secondary to taurine deficiency was reported in cats fed frozen whole ground raw rabbit (25). Additionally, analyses of various frozen whole-prey samples indicate possible insufficiencies in select essential AA (9). It is unknown how post-slaughter handling (i.e., dressing carcasses, grinding, freezer storage) affects nutrient concentration or availability of nutrients, including taurine. When an entire carcass is ground, taurine-rich organs and muscles are exposed to GIT (gastrointestinal tract) contents containing potentially taurine-degrading bacteria. Therefore, taurine concentrations could be affected by exposure to taurine-degrading GIT microbes and subsequent freezer storage. The purpose of this study was to evaluate the concentrations of essential AA in fresh, skinned ground rabbits with and without GIT and then determine if these concentrations are affected by freezing for 30 days. It was hypothesized that the presence of the GIT would reduce taurine concentrations and that these effects would be exacerbated by freezer storage.

## Materials and Methods

Experimental procedures were approved by the University of California-Davis Institutional Animal Care and Use Committee (Protocol #19040).

### Rabbits

Twenty-one freshly slaughtered 45- to 65-day-old Californian/New Zealand cross rabbit carcasses, raised with intention for sale as meat and consuming exclusively commercial rabbit pellets (A.L. Gilbert Farmer's Best Feed Rabbit Pellets), were purchased from a local producer (Penryn Rabbit Farm; Penryn, CA) in three groups of five and one group of six. The carcasses were each randomly assigned a number; placed into a sealed, water-tight plastic bag; and stored in a cooler on ice until same-day processing. Rabbit carcasses were individually weighed before they were skinned in the traditional manner (removal of pelt with ears and feet below carpal/tarsal joints) and then reweighed. Ten rabbits were eviscerated (removing the GIT and urinary bladder), then reweighed, and 10 rabbits were left intact. One additional rabbit was dissected for individual tissue samples.

### Processing and Analysis

Ten skinned, eviscerated rabbits (without GIT) and 10 skinned, intact rabbits (with GIT) were ground (including bones, head, internal organs) and mixed repeatedly using a Weston #22 meat grinder with 7 and 4.5 mm grinder plate until visually homogenous. The ground mixture from each rabbit was divided into two roughly equal portions by volume and placed into labeled, sealed commercial freezer bags. One of these was stored at −80°C for long-term preservation. Several sections were randomly removed from throughout the remaining bag (visually estimated to be about a palm-sized amount total), mixed, and checked for visual homogeneity. From this, aliquots (range 19.5–24.1 g) were distributed into duplicate labeled Falcon tubes. Three separated GITs with contents were homogenized as much as possible using a blender (after cutting the GITs into small pieces) and then sampled into aliquots as described above (range 16.2–17.1 g). In addition, entire organs (brain, heart, lung, liver, kidney) and thigh muscles were collected from a separate rabbit.

One set of samples was sealed with a cap and stored in a freezer (−18°C for 30 days; aliquots of 10 rabbits with and 10 without GIT in duplicate, and aliquots of three separated GIT in duplicate), and one set (as above plus entire organ samples) was kept for immediate processing.

For the first step of processing, homogenized sample dry matter (DM) was determined by freeze-drying the entire duplicate samples stored in Falcon tubes after overnight storage at −80°C using a SAVANT SpeedVac Concentrator SVC200H with Refrigerated Condensation Trap RT490) for 24–48 h until constant weight was achieved. Freeze-dried samples were then finely ground and well mixed using a standard coffee bean grinder. Total nitrogen (N) concentration of the freeze-dried samples (~1 g of each was submitted in a closed, labeled centrifuge tube) from fresh and frozen ground rabbits and individual organs of one additional rabbit (only 0.1 g submitted from these) was determined by the UC Davis Analytical Laboratory using AOAC Official Method 990.03: protein (crude) in animal feed, combustion method (26). Approximately 0.2 g was removed from each ground, freeze-dried sample for AA analysis. From this, three 0.05-g aliquots were removed. Analysis for 18 AA, including all essential feline AA except tryptophan, was performed on a Biochrom 30 AA Analyzer (Cambridge, UK) using methods described elsewhere (20) and including determination of methionine and cystine by use of performic acid oxidation with acid hydrolysis (AOAC Official Method 944.12: Amino acids in feeds) (27).

### Statistical Analysis

Statistical analysis was performed using computer software (Microsoft Excel 2011, Microsoft Corporation) and web-based applications (Social Science Statistics. Mann–Whitney *U* Test Calculator and Wilcoxon Signed Rank Test Calculator). Unpaired groups (rabbits with and without GIT) were compared using Mann–Whitney tests and paired groups (before and after freezing for 30 days) were compared using Wilcoxon signed rank tests. Probability of *p* ≤ 0.05 was accepted as statistically significant. Concentrations of AA are reported as both measured % DM and as g per 16 g N to allow for standardization relative to apparent protein concentration based on analyzed N.

## Results

The 20 rabbit carcasses collected in the present study had a median weight of 1.67 kg (range 1.34–2.23 kg). There was no difference in predressed body weights (BW) between rabbits left intact and those eviscerated (*p* = 0.67448; Table 1). The 10 removed GITs had a median weight of 410 g as is (range 282–496 g as is), which represented 21.1% of the total predressed BW (including pelt and feet; range 15.7–26.8%). Average of duplicate analyses of the 3 samples of only GIT plus contents showed relatively higher water content (79.4–79.7% water for fresh samples and 78.9–80.1% for frozen) and relatively low crude protein (CP) concentrations compared to samples of rabbits with or without GIT (36.9–39.% CP DM for fresh samples and 35.9–37.8% CP DM for frozen).

**Table 1:** Body weights of fresh carcasses and body water, nitrogen (N), and crude protein (CP) concentrations of ground rabbit samples analyzed fresh and after freezing for 30 days at −18°C, with and without gastrointestinal tracts plus contents (GIT); *n* = 10 each group.

	**Fresh**		**Frozen**	
	**Rabbits with GIT**	**Rabbits without GIT**	**Rabbits with GIT**	**Rabbits without GIT**
	**Median (range)**			
Weight, as is basis	1,675 (1,368–2,163)	1,671 (1,336–2,231)	–	–
Body water	73.85 (73.94–73.99)^*^	71.37 (69.20–72.56)^*^	73.76 (71.89–75.33)^*^	70.95 (69.11–72.86)^*^
% N, DM basis	9.21 (8.53–10.16)	10.05 (9.37–10.72)	9.00 (8.46–10.57)	10.01 (9.39–10.71)
% CP, DM basis	57.54 (53.31−63.5)^a^	62.82 (58.56–67.00)^a^	56.25 (52.88–66.06)^b^	62.57 (58.69–66.94)^b^

* *Significant comparisons of with and without GIT within each treatment of fresh vs. frozen (p < 0.05)*.

a *Significant comparisons between fresh and frozen samples with GIT (p < 0.05)*.

b *Significant comparisons between fresh and frozen samples without GIT (p < 0.05)*.

When AA concentrations of fresh ground rabbits with GITs were compared to those without GITs on a g/16 g N basis, 8 of 18 AA (glutamate, alanine, methionine, isoleucine, tyrosine, lysine, histidine, and arginine), five of which are essential, were greater in samples without GITs (Table 2; *p* < 0.05). However, when compared on a % DM basis, all 18 AA concentrations were higher in fresh samples without GITs, 15 of which were significantly greater (all except taurine, glycine, and cysteine; Table 3; *p* < 0.05). Taurine concentration did not greatly differ between groups with or without GITs and was only significantly higher in samples without GITs when expressed as % DM (*p* = 0.046).

**Table 2:** Amino acid concentrations of ground rabbit samples analyzed fresh and after freezing for 30 days at −18°C, with and without gastrointestinal tracts plus contents (GIT) and normalized for crude protein content; *n* = 10 each group.

	**Fresh**		**Frozen**	
**Amino acid**	**Rabbits with GIT**	**Rabbits without GIT**	**Rabbits with GIT**	**Rabbits without GIT**
	**g/16 g N; median (range)**			
Taurine	0.10 (0.075–0.145)	0.11 (0.087–0.129)	0.10 (0.088–0.147)	0.11 (0.094–0.125)
L-Asp	8.69 (7.49–9.36)	8.92 (8.43–9.95)	8.69 (8.08–9.47)	8.81 (8.10–9.37)
L-Thr	4.42 (3.32–5.32)^a^	4.68 (4.13–5.13)	4.66 (4.20–5.07)^a^	4.82 (4.32–5.41)
L-Ser	3.26 (2.02–4.36)^a^	3.59 (2.54–3.84)	3.81 (2.92–4.37)^a^	3.85 (3.54–4.12)
L-Glu	14.07 (12.46–15.22)^*^	14.77 (14.03–16.50)^*^^b^	13.91 (12.94–15.03)	14.29 (13.26–15.30)^b^
Gly	6.33 (5.96–7.41)^a^	6.36 (5.79–6.60)	5.66 (5.16–6.49)^a^	6.10 (5.58–6.47)
L-Ala	5.63 (5.46–6.21)^*^	5.87 (5.65–6.39)^*^	5.57 (5.21–6.10)	5.81(5.33–6.38)
L-Val	4.84 (4.04–4.99)	4.86 (4.56–5.47)	4.81 (4.32–5.18)	4.83 (4.39–5.51)
L-Cys	0.92 (0.70–1.41)^a^	1.02 (0.67–1.20)^b^	1.37 (1.02–1.69)^a^	1.58 (1.49–2.34)^b^
L-Met	2.16 (1.84–2.27)^*^	2.28 (2.17–2.57)^*^^b^	2.09 (1.96–2.31)^*^	1.94 (1.65–2.24)^*^^b^
L-Ile	4.00 (3.28–4.19)^*^	4.15 (3.91–4.66)^*^^b^	3.86 (3.56–4.16)	3.84 (3.41–4.08)^b^
L-Leu	7.71 (6.50–8.22)	8.01 (7.49–9.01)^b^	7.63 (6.96–8.25)	7.22 (6.53–7.94)^b^
L-Tyr	3.22 (2.65–3.38)^*^^a^	3.36 (3.18–3.78)^*^^b^	3.05 (2.81–3.18)^a^	2.98 (2.74–3.35)^b^
L-Phe	4.16 (3.54–4.48)	4.25 (3.99–4.75)^b^	3.93 (3.54–4.20)	3.90 (3.59–4.40)^b^
L-Lys	7.68 (6.41–8.22)^*^	8.14 (7.70–9.15)^*^^b^	7.34 (6.77–7.93)	7.64 (6.75–8.22)^b^
L-His	2.55 (2.15–2.78)^*^^a^	2.69 (2.48–2.99)^*^	2.39 (1.94–2.70)^a^	2.56 (2.25–2.91)
L-Arg	6.35 (5.93–7.01)^*^	6.76 (6.48–7.41)^*^^b^	6.27 (5.45–6.85)	6.32 (5.87–6.64)^b^
L-Pro	4.90 (4.70–5.44)^a^	4.97 (4.71–5.22)^b^	12.93 (4.34–15.06)^*^^a^	15.41 (13.86–16.28)^*^^b^

* *Significant comparisons of with and without GIT within each treatment of fresh vs. frozen (p < 0.05)*.

a *Significant comparisons between fresh and frozen samples with GIT (p < 0.05)*.

b *Significant comparisons between fresh and frozen samples without GIT (p < 0.05)*.

**Table 3:** Amino acid concentrations of ground rabbit samples analyzed fresh and after freezing for 30 days at −18°C, with and without gastrointestinal tracts plus contents (GIT) and normalized for water content; *n* = 10 each group.

	**Fresh**		**Frozen**	
**Amino acid**	**Rabbits with GIT**	**Rabbits without GIT**	**Rabbits with GIT**	**Rabbits without GIT**
	**% DM; median (range)**			
Taurine	0.05 (0.04–0.09)	0.07 (0.06–0.08)	0.05 (0.05–0.09)^*^	0.07 (0.06–0.07)^*^
L-Asp	4.91 (4.40–5.46)^*^	5.50 (5.35–6.01)^*^	4.85 (4.56–5.96)^*^	5.43 (5.05–6.20)^*^
L-Thr	2.55 (1.95–2.95)^*^^a^	2.98 (2.50–3.14)^*^	2.60 (2.34–3.20)^*^^a^	2.99 (2.75–3.58)^*^
L-Ser	1.85 (1.10–2.32)^*^^a^	2.27 (1.54–2.47)^*^	2.09 (1.63–2.56)^*^^a^	2.32 (2.14–2.67)^*^
L-Glu	8.03 (7.31–9.03)^*^	9.16 (8.79–9.97)^*^	7.86 (7.31–9.57)^*^	8.82 (7.95–10.12)^*^
Gly	3.63 (3.32–4.35)^a^	3.97 (3.39–4.36)	3.25 (2.87–3.73)^*^^a^	3.75 (3.39–4.10)^*^
L-Ala	3.27 (3.05–3.68)^*^	3.67 (3.44–3.93)^*^	3.16 (2.95–3.71)^*^	3.60 (3.40–3.97)^*^
L-Val	2.70 (2.37–2.92)^*^	3.04 (2.89–3.30)^*^	2.71 (2.44–3.24)^*^	2.99 (2.58–3.44)^*^
L-Cys	0.53 (0.41–0.75)^a^	0.64 (0.41–0.76)^b^	0.76 (0.57–1.11)^*^^a^	0.99 (0.92–1.38)^*^^b^
L-Met	1.22 (1.08–1.44)^*^	1.43 (1.35–1.56)^*^^b^	1.17 (1.11–1.52)	1.22 (0.97–1.34)^b^
L-Ile	2.24 (1.93–2.53)^*^	2.60 (2.43–2.81)^*^^b^	2.18 (2.01–2.68)	2.36 (2.06–2.69)^b^
L-Leu	4.39 (3.81–4.76)^*^	4.96 (4.80–5.45)^*^^b^	4.33 (3.94–5.16)	4.46 (3.83–5.19)^b^
L-Tyr	1.78 (1.55–2.06)^*^^a^	2.10 (2.04–2.29)^*^^b^	1.66 (1.59–2.05)^a^	1.84 (1.65–2.15)^b^
L-Phe	2.38 (2.08–2.62)^*^^a^	2.65 (2.57–2.87)^*^^b^	2.20 (2.00–2.63)^*^^a^	2.44 (2.12–2.84)^*^^b^
L-Lys	4.35 (3.76–4.92)^*^	5.05 (4.79–5.53)^*^^b^	4.12 (3.83–5.23)^*^	4.69 (3.96–5.44)^*^^b^
L-His	1.47 (1.26–1.66)^*^	1.71 (1.61–1.81)^*^	1.36 (1.11–1.58)^*^	1.60 (1.44–1.82)^*^
L-Arg	3.73 (3.37–4.20)^*^	4.22 (3.97–4.52)^*^^b^	3.57 (3.11–4.15)^*^	3.88 (3.49–4.40)^*^^b^
L-Pro	2.85 (2.60–3.35)^*^^a^	3.13 (2.76–3.45)^*^^b^	7.46 (2.37–8.83)^*^^a^	9.49 (8.60–10.17)^*^^b^

* *Significant comparisons of with and without GIT within each treatment of fresh vs. frozen (p < 0.05)*.

a *Significant comparisons between fresh and frozen samples with GIT (p < 0.05)*.

b *Significant comparisons between fresh and frozen samples without GIT (p < 0.05)*.

AA concentrations, water, and CP content, and fresh organ weights of kidney, thigh muscle, heart, brain, lung, and liver from the single additional rabbit are presented in Supplementary Table 1.

## Discussion

The presence or absence of the GIT strongly influenced many of the findings in the current study. The differential dilutional effect of the GIT on the results when normalized for CP simply reflects that the GITs contain much less protein overall compared to the entire carcass. In addition, the effect of the GITs with their contents suggests that they contain lower concentrations of most AA and/or differences in concentrations relative to total N. The contribution of nonprotein N present in GIT may lead to further overestimates of CP when using the standard conversion factor of 6.25. For example, although samples from hindgut fermenters, such as rabbits and horses, were not assessed, a previous study of cattle, pig, and chicken manure reported exact conversion values based on AA composition of 4.78–5.36 (28). In the present study, AA were also diluted on a DM basis in the samples with GIT. Although AA concentrations were always higher in samples without GITs, the specific AA that were apparently affected by GIT inclusion differed between the fresh and frozen groups. In the frozen group, three AA showed significant differences between samples with and without GIT (taurine, glycine, and cysteine), which were not apparent in fresh samples, and in the fresh group, four AA were significantly higher in samples without GITs but lost this effect after freezing (methionine, isoleucine, leucine, and tyrosine). This may reflect differential effects of freezer storage on individual AA or simply variability among samples. When normalized for protein content and compared on a g/16 g N basis, there were fewer differences in frozen vs. fresh samples. The AA concentrations of frozen samples with and without GITs were surprisingly stable; only methionine was lower and proline was higher in samples without GITs.

Freezing did not impact body water or CP content but appeared to have an effect on some AA when compared to fresh samples. Overall, cysteine, proline, glycine, and histidine followed the same pattern of change pre- and post-freezing in the samples with and without GITs, suggesting that these are likely affected by one or more aspects of handling or storage universal to all samples (i.e., freezing). However, threonine, serine, glycine, and histidine changed concentrations between fresh and frozen samples with GITs but not between those without, indicating some impact of the presence of the GIT and its contents. However, only threonine and histidine are essential AA. Surprisingly, seven other AA decreased in frozen samples of ground rabbit without GITs, indicating differential effects of freezing with or without the presence of the GIT. Likewise, when compared on % DM, for rabbits ground with GITs and for those without GITs, the findings were very similar except for a decrease in phenylalanine and no change in histidine for rabbits with GITs (Table 2). For those without GITs, the pattern of change was the same whether in g/16 g N or a percentage basis except that glutamate was not different.

Although glycine (on a g/16 g N basis) was also not different between groups with or without GITs when either fresh or frozen, it was higher in fresh samples with GITs compared to frozen. Glycine concentrations of gut contents are likely influenced by the exclusive conjugation of bile acids in the rabbit with this AA (29), depending on the presence and quantity of bile acids in the sample. The stability of glycine as a bile conjugate vs. when present in peptides and proteins has not been described in the literature to the author's knowledge, but it is possible that degradation of glycine in GIT contents during freezer storage could explain the differences seen in the present study. As already described, the apparent dilutional effect of GIT contents may also have played a role as especially evident on an absolute basis.

Although a pattern was not consistently obvious, it appeared that AA concentrations of rabbits with GITs trended toward the high end of the range for the rabbit with the smallest proportional GIT plus contents and trended toward the lower end of the range for the rabbit with the largest GIT plus contents (as % BW, DM basis). Assuming the majority of GIT weight is from contents, this further indicates a dilutional effect from the GIT contents, tempered by the AA concentrations of the GIT itself.

Taurine is a sulfur-containing beta-AA vital for maintaining normal retinal function (30), reproduction (31–33), and cardiac function (6). Cats obligatorily use taurine for bile acid conjugation (34), but have inadequate endogenous synthesis, making overall production insignificant compared to fecal losses (35). Many animal tissues (and presumably the evolutionary feline diet) contain high concentrations of taurine (20). Dietary taurine requirements in cats largely depend on the extent of losses associated with imperfect entero-hepatic recycling, which are further affected by microbial degradation in the gut as well as multiple dietary factors (10–20). Both the National Research Council (NRC) and the Association of American Feed Control Officials (AAFCO) recommend minimum taurine concentration for all feline life stages at 0.1% DM for extruded kibble diets; however, this is higher for canned diets (0.17 and 0.2% DM per NRC and AAFCO, respectively) (36, 37).

When compared to dietary concentration guidelines for all feline life stages per NRC and AAFCO (36, 37), all samples well exceeded the recommendations for all measured essential AA regardless of processing with or without GITs or whether measured fresh or post-freezing with the notable exception of taurine, which was an unexpected finding. In fact, median taurine concentrations in all four groups provided only 50–70% of the recommended 0.1% DM for extruded kibble diets and much lower compared with that for canned diets (0.17–0.2% DM). None of the whole ground rabbit samples met any recommendation for taurine concentrations for commercial diets (Table 2). Some samples would have met the NRC recommended minimum for “highly digestible purified diets” (0.04–0.053% DM depending on life stage) (37); however, these diets are typically only used in research settings, generally provide taurine in the crystalline form, and are likely of limited relevance to the needs of pet cats eating other types of diets. Although the requirement for taurine of cats consuming prey remains unknown, it seems unlikely that it would be less than that for commercial kibble diets due to the indigestible nature of certain components of the carcass. For example, dietary fiber plays a role in feline taurine needs (19), and several lines of evidence support that fur, collagen, or other indigestible components of prey carcasses function similarly to fiber (38–40). Further, diet type affects taurine metabolism (10, 11, 13, 18, 41), and previous studies in other species note differences in the gut microbial population with changes to feed texture (42, 43).

We had expected low taurine concentrations in rabbit GITs and that there would be, therefore, a strong effect of lower concentrations in rabbits ground with GITs when normalized for CP content, but this prediction was not supported by our findings. The ingredient list of the rabbit feed used by the producer did not include a source of purified taurine (Supplementary Table 2). Further, the primary components of rabbit feed are of plant origin and are not expected to contain taurine. We had also hypothesized that the presence of the GIT and exposure to taurine-degrading gut microbes would result in decreased concentrations in those samples; however, this was not the case. Perhaps prompt freezer storage was sufficient to interrupt microbial action, together with limited and controlled thawing time prior to analysis. This suggests that changes in other AA concentrations may occur by mechanisms other than microbial action or that there are other variables not yet elucidated.

Diet and age of rabbits also affect AA concentrations although effect on taurine was previously unknown (44, 45). A more recent study obtained rabbits (stillborn, 30–45 days of age, and >65 days of age) as frozen entire carcasses and then determined AA concentrations as well as AA digestibility in lyophilized, ground samples using the precision-fed cecectomized rooster assay (9). Those authors reported taurine concentrations that were well below or just meeting the lowest NRC and AAFCO recommendations for commercial feline diets (0.1% DM) in the 30- to 45- (0.01% DM) and the >65-day-old rabbits (0.1% DM); however, the stillborn rabbits, which came from a different source, were much higher in taurine content (0.29% DM) (9). The higher reported value for the stillborn rabbits is inconsistent with our findings (range 0.04–0.09% DM) as well as with previously published taurine concentrations for rabbits. Rabbits with only 0.06% taurine DM (measured from ground subsamples) were fed intact (not ground, not reported if previously frozen) to 10 cheetahs for 4 weeks, and normal serum taurine concentrations were maintained during this relatively short time (24); however, ideally, concurrent plasma and whole blood taurine measurements are preferred over serum due to potential variability in taurine content of serum secondary to sample-handling factors, such as clotting times and separation methods. Taurine deficiency leading to DCM was previously reported in 70% of 22 young domestic cats fed entire ground rabbit (unskinned, undressed) for a longer period of 10 months (25). The age of the rabbits and taurine concentrations were not reported although the authors did report low dietary vitamin E, which can contribute to taurine losses during processing (46). Subsequent publications by different authors cite a dietary concentration of 0.13% taurine DM for the diet used in the Glasgow et al. study (25), but the source of this value is unclear (9, 47). This concentration (0.13% DM) is greater than those documented in the current study (0.04–0.09% DM) as well as both AAFCO or NRC recommendations for dry extruded diets (0.1% DM), but it is lower than the recommended concentrations for canned diets (0.2% DM AAFCO; 0.17% DM NRC) (36, 37). It is unclear if the differences in measured taurine concentrations or outcomes among reports are due to variations in study length, small numbers of rabbit samples with natural interindividual variability, differences in feeding methods (intact vs. ground could affect taurine measurements or metabolism), direct vs. indirect determination of taurine concentrations, presence or absence of the pelt (possible fiber-like effects in the gut), age or source of rabbits or other factors.

It is known that certain organs contain more taurine than others (20). In order to investigate possible differential distribution among the various organs, single samples of selected organs were collected from one additional rabbit. Only the heart and lung (but not thigh muscle, brain, liver, or kidney) contained adequate concentrations of taurine compared to NRC and AAFCO recommendations (Supplementary Table 1). This was despite the high amount of CP, especially in thigh muscle (89% DM). Given that rabbits exclusively conjugate bile acids with glycine, it is not surprising the liver had low concentrations of taurine (<25% of thigh muscle, 10% of kidney or brain, and 1–3% of heart and lung concentrations). The myocardial necessity of taurine for normal metabolism is a likely reason for its high concentration in the heart; however, a similar statement cannot be made for lung. The finding of adequate taurine in these two organs was somewhat unexpected in the face of apparently low global taurine. We did not have multiple samples for comparison, and this is an area in need of further research.

In summary, the GIT of the rabbit, inclusive of its contents, can constitute a large percentage of rabbit BW and likely impacts the concentration of AA on both a compositional and dilutional basis although individual differences are difficult to elucidate given the variables and multifactorial nature of the effect. Freezing ground rabbit appears to impact the concentrations of only select AA (cysteine, proline, glycine, and histidine) independent of the presence or absence of the GIT and its contents. The marked increase in proline that occurred with freezing remains unexplained, especially because glutamate concentrations did not decrease (therefore, conversion of glutamate to proline is not the apparent mechanism); however, it is of limited practical significance given it is a nonessential AA. Regardless, changes in essential AA concentrations in this study due to freezing or processing would not be expected to have physiological consequences as all essential alpha-AA in all four groups remained in excess of NRC recommended allowances for all feline life stages. The effects of extended periods of freezer or refrigerator storage on AA concentrations, as may occur in some retail or household situations, remains unknown.

The low concentrations of taurine in rabbit, regardless of sample type and storage conditions, would very likely be physiologically significant if fed in large proportions or as the sole diet. It remains to be seen if there are differences in taurine concentrations between wild and domestically raised rabbits. It is possible that all lagomorphs are inherently low in taurine regardless of diet. This could result in negative clinical consequences if cats are fed unsupplemented rabbit exclusively or as a large proportion of the diet but may not be of consequence in a wild-type setting as cats do not consume rabbit exclusively in the wild even when they constitute a larger percentage of the diet (48). Perhaps the consumption of additional and varied prey species is sufficient to meet overall needs. In addition, it would be interesting to characterize AA concentrations in rabbit carcasses after industrial processing either for use in commercial pet food or for human consumption (including complete evisceration and removal of the head). Finally, assessment of rabbits of different breeds and from different producers would enable a wider perspective on the body composition of domestically raised rabbits.

It remains unknown if supplementation of taurine to concentrations recommended for either dry extruded or canned diets is sufficient to maintain normal taurine status of cats consuming whole-prey diets generally or rabbit specifically. Overall, the physiologic effect of whole-prey diets (and the various forms they can assume) remains uncharacterized and may be impacted by inclusion of the pelt or whether the prey is ground or intact. It is noteworthy that ecological studies have confirmed the consumption of rabbits or other lagomorphs by wild cats by examination of scat or stomach samples rather than direct observation; therefore, it is undocumented if cats consume rabbits with a particular pattern of preference and/or if this may vary with rabbit size (e.g., entire consumption of small/neonatal/young rabbits or only specific parts of larger rabbits).

## Data Availability Statement

The original contributions presented in the study are included in the article/Supplementary Material, further inquiries can be directed to the corresponding author/s.

## Ethics Statement

The animal study was reviewed and approved by University of California-Davis Institutional Animal Care and Use Committee (Protocol #19040).

## Author Contributions

JAL, AJF, and CCC conceived the study, wrote the grant, and provided logistical support and graduate student mentorship. TJO provided input on study design, arranged and conducted the experiment, and conducted the statistical analysis. All authors contributed to data interpretation and manuscript preparation.

## Conflict of Interest

TJO participates in continuing education events and other symposia sponsored or organized by Royal Canin, Hill's Pet Nutrition, and Nestlé; Purina PetCare. Various outreach programs and facilities within the Veterinary Medical College at which she works have been funded via donations from Nestlé; Purina PetCare, and Royal Canin. JAL is an investigator in clinical trials sponsored by Royal Canin and Nestlé; Purina PetCare. She develops educational materials for Brief Media, Mark Morris Institute, and Healthy Pet magazine. She participates as a speaker or attendee in continuing education events sponsored or organized by Royal Canin, Nestlé; Purina PetCare, and Hill's Pet Nutrition. AJF has advised Synergy Food Ingredients and has a grant from The Nutro Company. She participates in events and received remuneration for lectures or as an advisor to Nestlé; Purina PetCare, Mars Petcare, and the Pet Food and Mark Morris Institutes. The Veterinary Medical Teaching Hospital at the University of California, Davis received funding from Royal Canin to support a residency position, and from Nestlé; Purina PetCare to partially support a nutrition technician. A resident of the Nutrition Service received funds through the Hill's Pet Nutrition Resident Clinical Study Grants program. AJF and JAL collaborated on the resulting research project. The remaining author declares that the research was conducted in the absence of any commercial or financial relationships that could be construed as a potential conflict of interest.
