# Supplementary material for: Rabbit Carcasses for Use in Feline Diets: Amino Acid Concentrations in Fresh and Frozen Carcasses With and Without Gastrointestinal Tracts
Source: Front Vet Sci. 2021 Jan 21;7:592753. doi: 10.3389/fvets.2020.592753 (PMC7859488; doi:10.3389/fvets.2020.592753)
Supplement: Supplementary file 1 [file Data_Sheet_1.docx]

**Supplementary Table 1. Fresh weights, proportions of water and crude protein, and amino acid concentrations of selected freeze-dried, homogenized organs from a single rabbit**

|  | **Kidney** | **Thigh** | **Heart** | | **Brain** | **Lung** | **Liver** | **Kidney** | **Thigh** | **Heart** | **Brain** | **Lung** | **Liver** |
| --- | --- | --- | --- | --- | --- | --- | --- | --- | --- | --- | --- | --- | --- |
|  | **Organ weights, fresh as is basis (g)** | | | | | | |  | | | | | |
|  | **7.45** | **22.47** | | **5.18** | **3.18** | **10.60** | **7.45** |  |  |  |  |  |  |
|  | **% water** | | | | | | |  | | | | | |
|  | **77.2** | **76.68** | | **79.45** | **78.56** | **75.88** | **72.76** |  | | | | | |
|  | **% crude protein** | | | | | | |  | | | | | |
|  | **64.81** | **89.00** | | **69.44** | **51.88** | **58.63** | **66.31** |  | | | | | |
|  | **Amino acid concentrations** | | | | | | | | | | | | |
|  | **% DM** | | | | | | | **g/16 g N** | | | | | |
| **Tau** | 0.07 | 0.03 | 0.59 | | 0.08 | 0.19 | 0.01 | 0.11 | 0.03 | 0.86 | 0.15 | 0.33 | 0.01 |
| **L-Asp** | 5.13 | 8.12 | 5.96 | | 5.11 | 4.75 | 5.89 | 7.91 | 9.12 | 8.58 | 9.86 | 8.10 | 8.88 |
| **L-Thr** | 3.14 | 4.73 | 3.48 | | 3.01 | 2.97 | 3.36 | 4.85 | 5.31 | 5.01 | 5.80 | 5.06 | 5.06 |
| **L-Ser** | 2.97 | 3.85 | 2.90 | | 2.95 | 2.66 | 2.92 | 4.58 | 4.32 | 4.17 | 5.68 | 4.54 | 4.41 |
| **L-Glu** | 7.43 | 13.40 | 9.58 | | 7.46 | 6.90 | 8.81 | 11.47 | 15.06 | 13.79 | 14.37 | 11.76 | 13.29 |
| **Gly** | 2.92 | 3.85 | 4.09 | | 2.33 | 3.39 | 3.27 | 4.51 | 4.32 | 5.90 | 4.50 | 5.78 | 4.93 |
| **L-Ala** | 3.20 | 4.66 | 4.24 | | 2.97 | 3.50 | 3.54 | 4.93 | 5.24 | 6.10 | 5.73 | 5.97 | 5.33 |
| **L-Val** | 3.19 | 4.34 | 3.73 | | 2.99 | 3.53 | 3.88 | 4.92 | 4.88 | 5.37 | 5.77 | 6.03 | 5.85 |
| **L-Cys** | 1.07 | 0.59 | 1.23 | | 1.07 | 0.99 | 1.12 | 1.65 | 0.67 | 1.77 | 2.06 | 1.70 | 1.70 |
| **L-Met** | 1.31 | 2.46 | 1.44 | | 1.20 | 0.81 | 1.57 | 2.02 | 2.76 | 2.07 | 2.32 | 1.39 | 2.37 |
| **L-Ile** | 2.34 | 3.94 | 2.62 | | 2.11 | 1.63 | 2.87 | 3.61 | 4.43 | 3.77 | 4.07 | 2.79 | 4.33 |
| **L-Leu** | 5.13 | 7.07 | 5.88 | | 4.73 | 5.27 | 6.03 | 7.92 | 7.94 | 8.47 | 9.13 | 8.99 | 9.10 |
| **L-Tyr** | 2.24 | 3.12 | 2.47 | | 2.11 | 2.05 | 2.51 | 3.45 | 3.50 | 3.55 | 4.08 | 3.50 | 3.79 |
| **L-Phe** | 2.81 | 3.60 | 3.10 | | 2.78 | 2.87 | 3.41 | 4.34 | 4.04 | 4.47 | 5.35 | 4.90 | 5.15 |
| **L-Lys** | 4.19 | 0.74 | 5.20 | | 3.91 | 4.56 | 4.63 | 6.47 | 0.83 | 7.49 | 7.54 | 7.77 | 6.99 |
| **L-His** | 1.54 | 2.42 | 1.91 | | 1.63 | 2.17 | 1.84 | 2.37 | 2.72 | 2.75 | 3.15 | 3.70 | 2.77 |
| **L-Arg** | 3.80 | 5.90 | 4.48 | | 3.36 | 3.42 | 4.26 | 5.89 | 6.63 | 6.45 | 6.48 | 5.84 | 6.43 |
| **L-Pro** | 2.63 | 3.29 | 3.40 | | 2.27 | 2.76 | 2.90 | 4.06 | 3.69 | 4.89 | 4.38 | 4.71 | 4.38 |

**Supplementary Table 2: Ingredient and selected calculated nutrient composition of A.L. Gilbert Farmer’s Best Feed Rabbit Pellets, as fed basis**

| Ingredients: Suncured Alfalfa Pellets, Wheat Millrun, Soybean Meal, Cane Molasses, Whole Wheat, Dried Whey, Salt; Soybean Oil, Corn Syrup, Corn Distillers Dried Grains With Solubles, Glycerin; Vegetable Oil, Magnesium Chloride, Dicalcium Phosphate, Monocalcium Phosphate, Hydrochloric Acid, Sodium Bisulfite, Natural and Artificial Flavors, Potassium Sulfite, Sodium Sulfite, Copper Sulfate, Extracted Streptomyces Meal, Condensed Fermentation Corn Extractives, Riboflavin Supplement, Vitamin A Supplement, Vitamin D3 Supplement, Biotin, Vitamin B-12 Supplement, Choline Chloride, Corn Cob Fractions, Zinc Sulfate, Calcium Pantothenate, Niacin Supplement, Vitamin E Supplement, Calcium Carbonate, Manganous Oxide, Ferrous Sulfate, Calcium Iodate, Cobalt Carbonate, Ground Limestone, Sodium Selenite. | | |
| --- | --- | --- |
| **Component** | **Units** | **Concentration** |
| Dry matter | % | 89.37 |
| Crude protein | % | 16.47 |
| Crude fat | % | 2.65 |
| Crude fiber | % | 18.50 |
| Acid detergent fiber | % | 23.26 |
| Neutral detergent fiber | % | 37.84 |
| Ash | % | 8.10 |
| Calcium | % | 0.87 |
| Phosphorus | % | 0.47 |
| Sodium | % | 0.33 |
| Chloride | % | 0.61 |
| Magnesium | % | 0.29 |
| Potassium | % | 1.88 |
| Sulfur | % | 0.26 |
| Cobalt | mg/kg | 0.24 |
| Copper | mg/kg | 3.81 |
| Iodine | mg/kg | 0.16 |
| Iron | mg/kg | 65.04 |
| Manganese | mg/kg | 12.93 |
| Selenium | mg/kg | 0.06 |
| Zinc | mg/kg | 42.16 |
| Vitamin A | IU/kg | 1136.99 |
| Vitamin D | IU/kg | 252.59 |
| Vitamin E | IU/kg | 2.27 |
| Biotin | mg/kg | 0.002 |
| Niacin | mg/kg | 0.005 |
| Lysine | % | 0.73 |
| Methionine | % | 0.23 |
|  |  |  |
